# Outcomes of Patients Undergoing Rotational Atherectomy with Intra-Aortic Balloon Pump Support in Patients with Multivessel Disease and Low Left Ventricular Ejection Fraction

**DOI:** 10.31083/j.rcm2401027

**Published:** 2023-01-13

**Authors:** Hao Hu, Zhiqing Guo, Jiawei Wu, Likun Ma

**Affiliations:** ^1^Department of Cardiology, The First Affiliated Hospital of USTC, Division of Life Sciences and Medicine, University of Science and Technology of China, 230001 Hefei, Anhui, China; ^2^Department of Cardiology, The Affiliated Provincial Hospital of Anhui Medical University, 230001 Hefei, Anhui, China

**Keywords:** rotational atherectomy, coronary artery disease, intra-aortic balloon pump, prognosis

## Abstract

**Background:**

The aim of the present study was to investigate whether intra-aortic balloon pump (IABP) support was associated with better outcomes after rotational atherectomy (RA) in patients with multivessel disease and low left ventricular ejection fraction (LVEF).

**Methods:**

Between January 2015 and December 2021, 596 consecutive patients with severely calcified coronary lesions who underwent elective RA were retrospectively enrolled. Of these, a total of 156 patients were included in this study based on the propensity score matching and divided into two groups according to elective IABP insertion (IABP group, n = 80) or no insertion (non-IABP group, n = 76) before the RA procedure. The primary endpoints were procedural success and major adverse cardiovascular events (MACE) before discharge. The secondary endpoints were mortality and readmission due to heart failure (HF) during 90-day and 180-day follow-up.

**Results:**

77 of patients (96.3%) in the IABP group and 72 of patients (94.7%) in the non-IABP group got procedural success (*p* = 0.714), separately. We had not observed significant differences in periprocedural complications except for less frequent hypotension in the IABP group (*p *< 0.001). In-hospital MACE occurred in 7.5% of patients who received IABP support, which was significantly lower compared to the non-IABP group (*p* = 0.002). In addition, the cumulative incidence of readmission due to HF was also significantly lower in the IABP group during the 90-day (*p *< 0.001) and 180-day (*p* = 0.004) follow-up. However, there were no significant differences between groups regarding the incidence of all-cause mortality.

**Conclusions:**

The present study suggests the important role of IABP support in improving the outcomes of patients after RA if multivessel disease and low LVEF are anticipated. Prophylactic IABP implantation was related to a lower incidence of in-hospital MACE, and readmission due to HF within 90-day and 180-day follow-up without significant impact on the procedural success and all-cause mortality.

## 1. Introduction

Moderate or severe calcified coronary lesions occurred in approximately 20% to 38% of cases in patients who underwent percutaneous coronary intervention (PCI) [1, 2]. Although Rotational atherectomy (RA) is recommended to process heavily calcified lesions by American Heart Association 2011 guidelines for PCI [3]. Worse cardiovascular outcomes including significant mortality rates after RA are noted in patients with multivessel disease and impaired left ventricular (LV) function [4]. These patients have poor reserve to withstand the consequences of ischemia resulting from RA procedures. Hypotension, heart failure, and even cardiogenic shock (CS) may often occur in these patients.

The role of intra-aortic balloon pump (IABP) in augmenting coronary blood flow, decreasing myocardial oxygen demand, and maintaining hemodynamic stability is established. Additionally, IABP was uniquely effective in the treatment of cardiogenic shock complicating acute myocardial infarction (AMI). Nevertheless, the strategy of routine IABP placement before PCI (prophylactic IABP) in high-risk and complex coronary lesions is still controversial [5, 6], and its influence on the in-hospital and short-term outcomes following RA has not been well evaluated.

Therefore, the current study was carried out to assess the potential usefulness of IABP support to improve clinical outcomes after RA in patients with multivessel disease and reduced left ventricular ejection fraction (LVEF).

## 2. Methods

### 2.1 Study Population

**Fig. 1.**
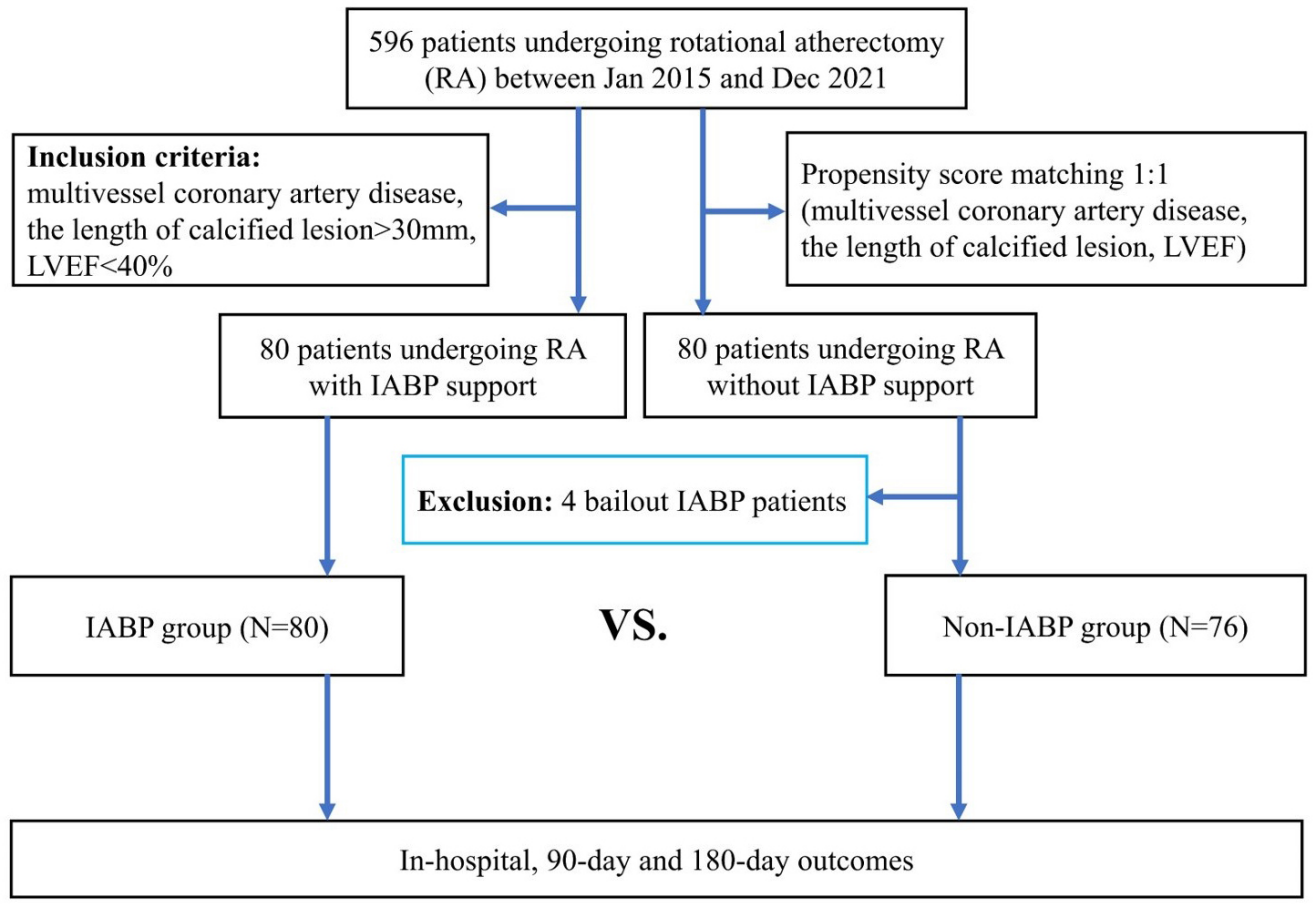
**Study flow chart**. Abbreviation: RA, rotational atherectomy; LVEF, left ventricular ejection fraction; IABP, intra-aortic balloon pump.

Between January 2015 and December 2021, 579 consecutive patients who received RA therapy for severely calcified coronary lesions were retrospectively screened in our institution. Inclusion criteria were as following: (1) The length of calcified lesions >30 mm; (2) Multivessel coronary artery disease (CAD) with ≥70% diameter stenosis; (3) LVEF <40%. Patients who were hemodynamically unstable, presenting with ST-segment elevation myocardial infarction (STEMI), or patients refused to receive RA were excluded. Finally, IABP was inserted in 80 of the 596 patients before the RA procedure, and they were included in the IABP group. Analysis of propensity score matching (PSM) was applied to reduce the potential effect of bias based on propensity score of each patient. After PSM, 160 patients undergoing RA (80 patients in each study group) were matched in the field of multivessel coronary disease, the length of calcified lesions >30 mm, and LVEF. 4 patients who received a bailout IABP implantation were excluded. Finally, 156 patients were included in the present study, of whom 80 were in the IABP group and 76 were in the non-IABP group, separately (Fig. 1). The Institutional Review Board approved the data collection procedure of the study and all participants signed informed consent before RA procedure.

### 2.2 Procedural Details

All RA procedures were performed by three senior experienced interventional cardiologists with the Rotablator system (Boston Scientific Corporation, Natick, MA, USA). The arterial access site was chosen based on peripheral vascular conditions and procedural requirements. Initial RA burr size was either 1.25 mm, 1.5 mm, or rarely 1.75 mm according to senior operators’ selection, then the burr was advanced proximally to the lesion, and moved forward with a slow pecking motion. The initial burr speed was set within the range from 140,000 to 180,000 rpm with the duration of each run less than 30 s, and a decrease in rotational speed >5000 rpm was carefully avoided. To reduce the occurrence of slow flow/no reflow, a pressured Rota-flush solution consisting of heparin, verapamil, and nitroglycerin was continuously infused into the coronary artery through a 4Fr Teflon sheath of the Rotablator system. An independent experienced cardiologist assessed the presence of slow-flow/no re-flow phenomenon by injecting a sufficient contrast medium immediately after the ablation pass. Following RA, routine balloon predilation to facilitate Drug-eluting stents (DES) implantation was performed. The IABP was placed percutaneously via the femoral artery, and 1:1 electrocardiographic triggering was initiated before starting the RA. Before removal of IABP, the electrocardiographic triggering was gradually down regulated from 1:1 to 1:2 to 1:3. The time to remove IABP was mainly determined by the patient’s clinical status (usually 4 to 24 hours following PCI). The decision to insert an IABP was left to the discretion and guidance of the supervising cardiologists. All patients received pretreatment with 300 mg aspirin and a loading dose of P2Y12 inhibitor (clopidogrel or ticagrelor) prior to RA, as well as the secondary prevention of CAD after the procedure. Cardiac biomarkers (Troponin I) were measured before PCI, and 6, 12, and 24 h after the RA procedure.

### 2.3 Definitions

Severely calcified lesions were either visually assessed by coronary angiography, defined as radiopacities noted without cardiac motion before contrast injection, or Intra-vascular ultrasound (IVUS) indicated superficial calcium involving more than 3 quadrants. Planned RA was defined as RA performed directly before balloon predilation, while bailout RA was RA performed after failure to balloon predilation or stents deliver to target lesions. Slow flow/no re-flow was defined as less than Thrombolysis in Myocardial Infarction (TIMI) III flow grade in the absence of dissection or thrombus immediately after RA. A final residual stenosis <30% complied with TIMI flow grade III after stents placement was considered procedural success. The procedure was considered a failure if patients received emergent coronary artery bypass grafting (CABG) and/or PCI, or other severe RA-related complications (death, coronary perforation) developed before discharge. Periprocedural myonecrosis was defined as troponin I above threefold of the upper limit of normal or a 50% increase from the baseline level [7].

### 2.4 Follow-Up and Endpoints

All patients were closely followed at 90-day and 180-day intervals after discharge. Follow-up information was obtained by clinicians through outpatient clinic visits, phone interviews, and hospital medical records. The primary endpoints of the present study included procedural success, and in-hospital major adverse cardiovascular events (MACE). MACE consisted of cardiac death, heart failure, target vessel revascularization (TVR), and stent thrombosis (ST). Unless a non-cardiac origin was surely documented, death was considered to be cardiac in origin. Deterioration in signs and symptoms of in patients with previous chronic heart failure (CHF) or new-onset heart failure (HF) requiring urgent therapy was considered as in-hospital HF. Diagnostic criteria was based on an intravenous administration of diuretic drugs, vasodilators, or inotropic drugs, and including at least one of the followings: cardiac pulmonary edema or pulmonary vascular congestion on chest radiograph; rales >one-third of the lung fields due to HF; left ventricular end-diastolic pressure (LVEDP) >18 mmHg; or dyspnea, with a Po2 <80 mmHg or an oxygen saturation <90% without oxygen inhaled (significant lung disease excepted). TVR was defined as any repeat PCI or CABG of the target vessel due to stent thrombosis or perforation. Thrombus of the target lesion on either angiography or autopsy examination was considered as ST according to the Academic Research Consortium [8]. The secondary endpoints consisted of all-cause mortality and readmission due to HF at 90- and 180-day intervals after discharge. Readmission due to HF was defined as readmission primarily for the treatment of HF needing the use of intravenous therapy such as diuretics, inotropic agents, or vasodilators.

### 2.5 Statistical Methods

The SPSS 26.0 system (IBM, Armonk, NY, USA) was utilized for statistical calculations. A logistic model was used to calculate the probability of receiving a IABP support before RA procedure (the propensity score). Baseline characteristics including age, male, hypertension, diabetes mellitus (DM), atrial fibrillation (AF), history of HF, pre-MI, pre-PCI, chronic kidney disease (CKD), LVEF, NT-proBNP, systolic blood pressure (SBP) before RA, target vessel (LAD, LCX, or RCA), and diseased vessels (two or three) were set as covariates. Based on the propensity score in a 1:1 (IABP:Non-IABP) fashion, the nearest neighbor matching was performed with a maximum caliper of 0.2. Categorical variables were reported as value (percentage) and Chi-squared or Fisher’ exact test was utilized. If the continuous variables were normally distributed determined by the Wilk-Shapiro test, they were reported as a mean ± SD, and intergroup differences were compared using an unpaired Student’s *t* test. Otherwise, non-normal distribution data was shown as median [25th–75th quartiles], and intergroup differences were compared using a Mann-Whitney U test. In addition, we compared the cumulative incidence of all-cause mortality, 90-day and 180-day readmission due to HF using the Kaplan-Meier method and the log-rank test. To identify the influential factors for 90-day and 180-day readmission due to HF, a Cox regression model was performed. All reported *p* values were 2 tailed, and intergroup differences were considered statistically significant when the probability was <0.05.

## 3. Results

### 3.1 Baseline Clinical Characteristics

Baseline demographics, comorbidities, and results of laboratory test were presented in Table 1. More frequent history of prior MI (26.3% vs. 7.9%, *p* = 0.002), more often CHF (42.5% vs. 5.3%,* p <* 0.001), higher level of low density lipoprotein-cholesterol (LDL-C) (2.1 ± 0.8 mmol/L vs. 1.8 ± 0.6 mmol/L, *p* = 0.036) and NT-proBNP [1024.0 (201.3–2684.3) pg/mL vs. 284.0 (75.8–904.5) pg/mL, *p *< 0.001] were observed in the IABP group. Fewer patients in the IABP group received nitrates and calcium channel blockers than that in the non-IABP group. However, no significant difference was observed with regard to other comorbidities, laboratory test results, and medications. Vital signs including baseline pressure and heart rates (HR), were also comparable.

**Table 1.** **Baseline patient characteristics**.

Variables		All (*n* = 156)	Non-IABP group (*n* = 76)	IABP group (*n* = 80)	*p*-value
Age (years)		72.3 ± 8.9	72.8 ± 8.9	71.9 ± 9.2	0.544
Male, *n* (%)		94 (60.3)	49 (64.5)	45 (56.3)	0.294
Hypertension,* n* (%)		120 (76.9)	62 (81.6)	58 (72.5)	0.179
Diabetes mellitus, *n* (%)		60 (38.5)	33 (43.4)	27 (33.8)	0.215
Atrial fibrillation, *n* (%)		16 (10.3)	6 (7.9)	10 (12.5)	0.343
Smoking, *n* (%)		56 (35.9)	28 (36.8)	28 (35.0)	0.811
Heart failure,* n* (%)		38 (24.4)	4 (5.3)	34 (42.5)	<0.001
LVEF (%)		33.8 ± 1.4	34.0 ± 1.4	33.6 ± 1.3	0.067
CKD, *n* (%)		7 (4.5)	3 (3.9)	4 (5.0)	1.000
Dialysis, *n* (%)		2 (1.3)	1 (1.3)	1 (1.3)	1.000
Pre-MI, *n* (%)		27 (17.3)	6 (7.9)	21 (26.3)	0.002
Pre-PCI, *n* (%)		62 (39.7)	28 (36.8)	34 (42.5)	0.470
Stroke, *n* (%)		52 (33.3)	25 (32.9)	27 (33.8)	0.910
Medication, *n* (%)					
	ACEI/ARB	78 (50.0)	41 (53.9)	37 (46.3)	0.337
	CCB	45 (28.8)	31 (40.8)	14 (17.5)	0.001
	Nitrates	76 (48.7)	44 (57.9)	32 (40.0)	0.025
	β-blocker	91 (58.3)	43 (56.6)	48 (60.0)	0.665
	Statins	154 (98.7)	76 (100.0)	78 (97.5)	0.497
	Aspirin	156 (100.0)	76 (100.0)	80 (100.0)	-
	Clopidogrel	78 (50.0)	43 (56.6)	35 (43.8)	0.109
	Ticagrelor	78 (50.0)	33 (43.4)	45 (56.3)	0.109
TC (mmol/L)		3.8 ± 1.1	3.7 ± 0.9	3.9 ± 1.1	0.081
TG (mmol/L)		1.4 ± 0.7	1.4 ± 0.6	1.4 ± 0.7	0.803
LDL-C (mmol/L)		1.9 ± 0.8	1.8 ± 0.6	2.1 ± 0.8	0.036
HDL-C (mmol/L)		1.1 ± 0.3	1.0 ± 0.3	1.1 ± 0.3	0.719
Creatinine (umol/L)		75 (61.0–90.0)	73.0 (59.0–90.0)	76.0 (63.0–94.5)	0.215
NT-proBNP (pg/mL)		568.5 (118.0–1453.1)	284.0 (75.8–904.5)	1024.0 (201.3–2684.3)	<0.001
SBP before RA (mmHg)		137.4 ± 22.5	138.9 ± 21.2	135.9 ± 23.6	0.401
DBP before RA (mmHg)		71.2 ± 12.4	71.5 ± 12.3	70.9 ± 12.6	0.783
HR before RA (bpm)		76.6 ± 13.9	74.9 ± 13.3	78.3 ± 14.4	0.133
Admission to procedure (days)		3.1 ± 0.4	3.0 ± 0.4	3.1 ± 0.5	0.112

LVEF, left ventricular ejection fraction; CKD, chronic kidney disease; MI, myocardial infarction; PCI, percutaneous coronary intervention; ACEI, angiotensin-converting enzyme inhibitor; ARB, angiotensin receptor blocker; CCB, calcium channel blocker; TC, total cholesterol; TG, triglyceride; LDL-C, low density lipoprotein-cholesterol; HDL-C, high density lipoprotein-cholesterol; SBP, systolic blood pressure; DBP, diastolic blood pressure; HR, heart rates; IABP, Intra-aortic balloon pump.

### 3.2 Angiographic and Procedural Details

Table 2 showed angiographic and procedural characteristics. The incidence of procedural success was similar (94.7% vs. 96.3%, *p* = 0.714) in the two groups. Of note, higher post-procedural SBP was observed in the IABP group (112.7 ± 22.5 mmHg vs. 94.3 ± 14.8 mmHg, *p *< 0.001), there was no significant difference in the incidence of vasopressors usage in the IABP and non-IABP group (8.8% vs. 11.8%, *p* = 0.525). Moreover, lesion and other procedural characteristics showed no significantly difference between the two groups.

**Table 2.** **Angiographic and procedural characteristics**.

Variables		All (*n* = 156)	Non-IABP group (*n* = 76)	IABP group (*n* = 80)	*p*-value
Target vessel, *n *(%)					
	LAD	136 (87.2)	65 (85.5)	71 (88.8)	0.547
	LCX	5 (3.2)	3 (3.9)	2 (2.5)	0.676
	RCA	15 (9.6)	8 (10.5)	7 (8.8)	0.707
Diseased vessels, *n* (%)					0.245
	Two	31 (19.9)	18 (23.7)	13 (16.2)	
	Three	125 (80.1)	58 (76.3)	67 (83.8)	
Reference diameter (mm)		2.86 ± 0.38	2.87 ± 0.39	2.85 ± 0.38	0.724
MLD (mm)		0.49 ± 0.31	0.54 ± 0.31	0.45 ± 0.30	0.078
Stenosis, %		82.3 ± 12.7	80.9 ± 10.8	83.7 ± 14.2	0.175
Lesion length (mm)		36.3 ± 7.3	36.3 ± 7.9	36.4 ± 6.7	0.933
Angulation >45°, *n* (%)		86 (55.1)	45 (59.2)	41 (51.2)	0.318
Primary RA, *n* (%)		101 (64.7)	50 (65.8)	51 (63.7)	0.790
Burr number, *n* (%)					0.395
	1	145 (92.9)	72 (94.7)	73 (91.3)	
	2	11 (7.1)	4 (5.3)	7 (8.8)	
Final burr size, *n* (%)					
	1.25 mm	39 (25.0)	16 (21.1)	23 (28.7)	0.267
	1.5 mm	108 (69.2)	56 (73.7)	52 (65.0)	0.240
	1.75 mm	9 (5.8)	4 (5.3)	5 (6.3)	1.000
Total run time (s)		42.0 (30, 65.5)	38.5 (27.2, 63)	45.0 (33.5, 66.8)	0.110
Mean rotational speed (×10,000 rpm)		15.2 ± 1.52	15.2 ± 1.54	15.1 ± 1.51	0.958
Rotablations times		3.9 ± 2.1	3.7 ± 2.2	4.0 ± 2.1	0.338
IVUS guided, *n* (%)		20 (12.8)	9 (11.8)	11 (13.8)	0.722
SBP in RA (mmHg)		103.1 ± 21.2	94.3 ± 14.8	112.7 ± 22.5	<0.001
DBP in RA (mmHg)		66.2 ± 15.2	65.8 ± 16.3	66.5 ± 14.1	0.764
HR in RA (bpm)		68.8 ± 14.8	67.6 ± 15.5	70.1 ± 14.1	0.320
Vasopressor usage *n* (%)		16 (10.3)	9 (11.8)	7 (8.8)	0.525
Procedural success *n* (%)		149 (95.5)	72 (94.7)	77 (96.3)	0.714

LAD, left anterior descending artery; LCX, left circumflex artery; RCA, right coronary artery; MLD, minimal luminal diameter; IVUS, intravascular ultrasound; RA, rotational atherectomy; SBP, systolic blood pressure; DBP, diastolic blood pressure; HR, heart rates.

### 3.3 In-Hospital, 90-Day, and 180-Day Outcomes

Table 3 summarized outcomes of in-hospital, 90-day and 180-day follow-up. Clinical follow-up was accomplished in all cases. Hypotension was less frequently observed in the IABP group (13.8% vs. 53.9%, *p *< 0.001), and there was a trend towards less frequent slow flow/no re-flow (35.0% vs. 46.1%, *p* = 0.160) in this group. Other periprocedural complications including bradycardia, complete atrioventricular block, dissection, perforation, and coronary spasm were not significantly different in the two groups. No patients developed sinus arrest and burr entrapment in this study. The admission days were significantly shorter in the IABP group than in the non-IABP group (5.6 ± 1.0 vs. 7.1 ± 2.9, *p *< 0.001).

**Table 3.** **In-hospital and follow-up outcomes [*n* (%)]**.

Variables		All (*n* = 156)	Non-IABP group (*n* = 76)	IABP group (*n* = 80)	*p*-value
Periprocedural complications					
	Slow flow/no re-flow	63 (40.4)	35 (46.1)	28 (35.0)	0.160
	Hypotension	52 (33.3)	41 (53.9)	11 (13.8)	<0.001
	Bradycardia	29 (18.6)	14 (18.4)	15 (18.8)	0.958
	Complete AV block	1 (0.6)	1 (1.3)	0 (0)	0.487
	Sinus Arrest	0 (0)	0 (0)	0 (0)	-
	Dissection	24 (15.4)	14 (18.4)	10 (12.5)	0.306
	Perforation	6 (3.8)	3 (3.9)	3 (3.8)	1.000
	Burr entrapment	0 (0)	0 (0)	0 (0)	-
	Coronary spasm	52 (33.3)	28 (36.8)	24 (30.0)	0.365
In-hospital outcomes					
	MACE	26 (16.7)	20 (26.3)	6 (7.5)	0.002
	Heart failure	23 (14.7)	18 (23.7)	5 (6.3)	0.002
	ST	0 (0)	0 (0)	0 (0)	-
	TLR	4 (2.6)	2 (2.6)	2 (2.5)	1.000
	Death	4 (2.6)	3 (3.9)	1 (1.3)	1.000
	Periprocedural myonecrosis	48 (30.8)	26 (34.2)	22 (27.5)	0.364
	Admission days	6.3 ± 2.2	7.1 ± 2.9	5.6 ± 1.0	<0.001
Outcomes within 90-day follow up					
	Readmission	37 (23.7)	28 (36.8)	9 (11.3)	<0.001
	All-cause mortality	21 (13.5)	13 (17.1)	8 (10.0)	0.194
Outcomes within 180-day follow up					
	Readmission	43 (27.6)	29 (38.2)	14 (17.5)	0.004
	All-cause mortality	22 (14.1)	14 (18.4)	8 (10.0)	0.131

AV, atrioventricular; MACE, major adverse cardiovascular events; ST, stent-thrombosis; TLR, target lesion revascularization.

Compared to the non-IABP group, in-hospital MACE was less frequently observed in the IABP group (7.5% vs. 26.3%, *p* = 0.002), mainly driven by in-hospital HF (6.3% vs. 23.7%, *p* = 0.002), as shown in Table 3. Compared to the non-IABP group, the incidence of periprocedural myonecrosis tended to be lower (27.5% vs. 34.2%, *p* = 0.364). No significant difference as for cardiac death and TVR were observed between the two groups, and stent thrombosis was observed in neither group.

**Fig. 2.**
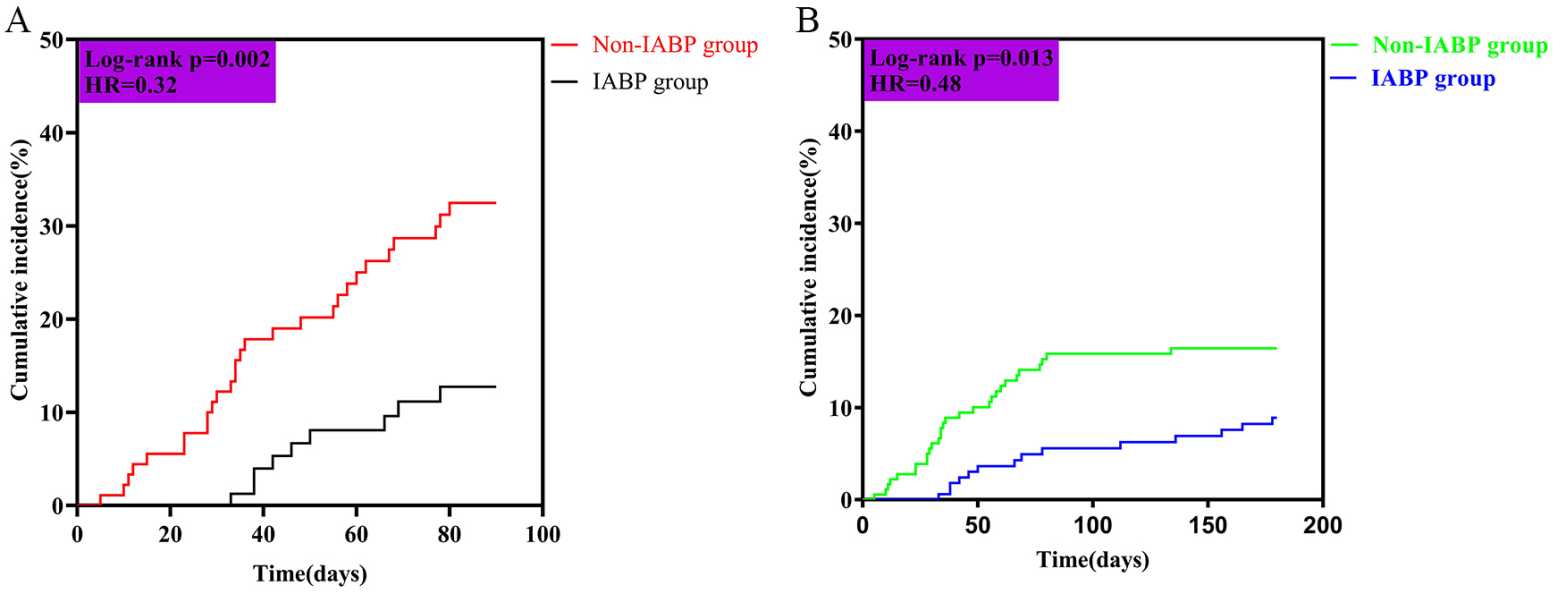
**Kaplan-Meier curves estimate incidence of readmission due to HF for patients undergoing elective RA with and without IABP support**. (A) Kaplan-Meier curves of cumulative incidence of readmission due to HF within 90-day follow-up. (B) Kaplan-Meier curves of cumulative incidence of readmission due to HF within 180-day follow-up. Abbreviations: IABP, intra-aortic balloon pump; HF, heart failure; RA, rotational atherectomy; HR, hazard ratio.

The Kaplan-Meier analysis showed a significantly lower incidence of readmission due to HF in the IABP group during the 90-day follow-up (log-rank test: *p* = 0.002, HR = 0.32, 95% CI: 0.17–0.61, Fig. 2A).

In addition, the incidence was also significantly lower (log-rank test: *p* = 0.013, HR = 0.48, 95% CI: 0.27–0.88, Fig. 2B) during the 180-day follow-up.

**Fig. 3.**
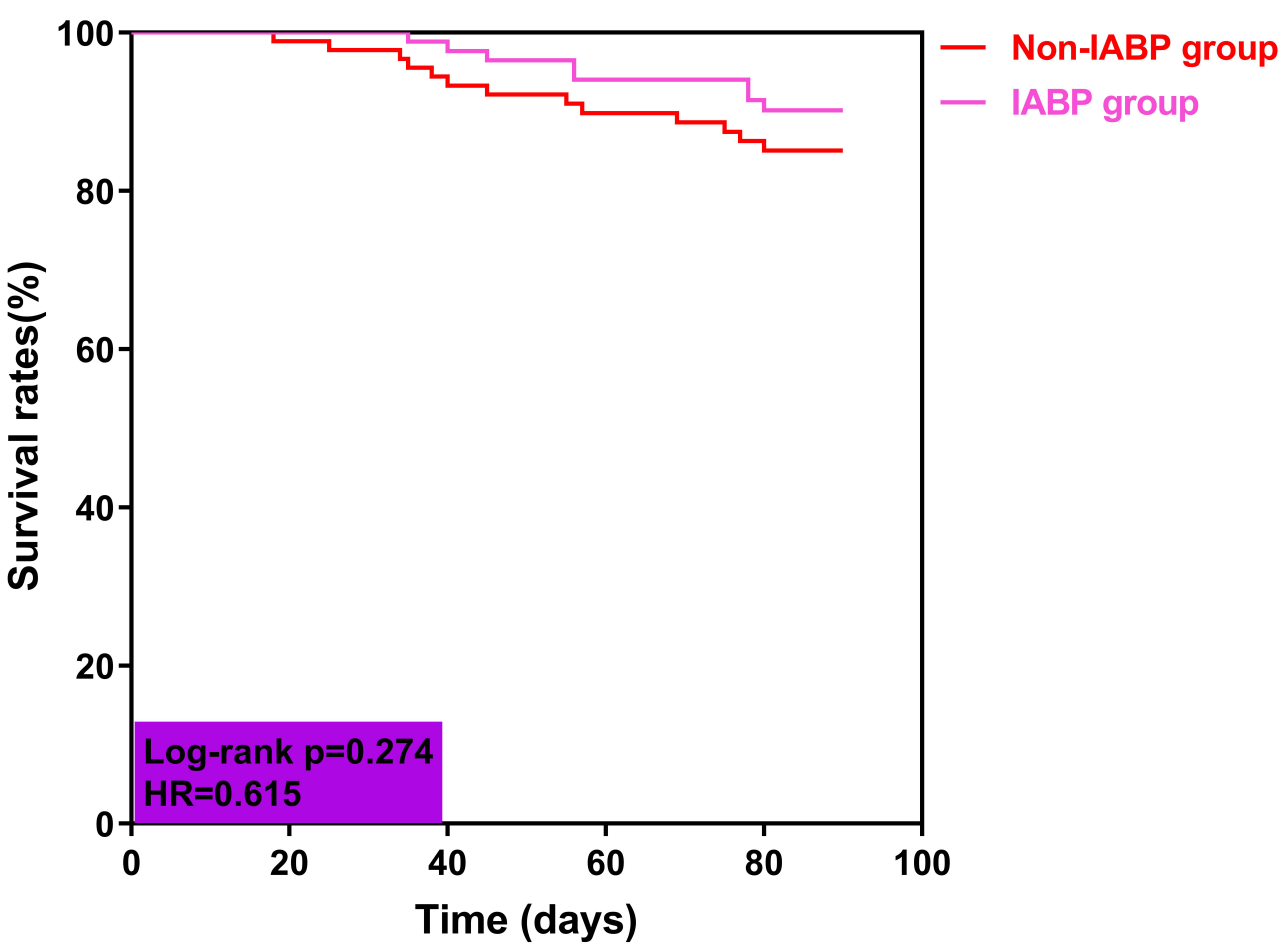
**Kaplan-Meier curves for cumulative survival rates within 90-day follow-up**. Abbreviations: HR, hazard ratio; IABP, intra-aortic balloon pump.

Furthermore, Kaplan-Meier analysis showed that the cumulative survival rates within 90-day follow up were not different between the two groups (*p* = 0.274, Fig. 3).

**Fig. 4.**
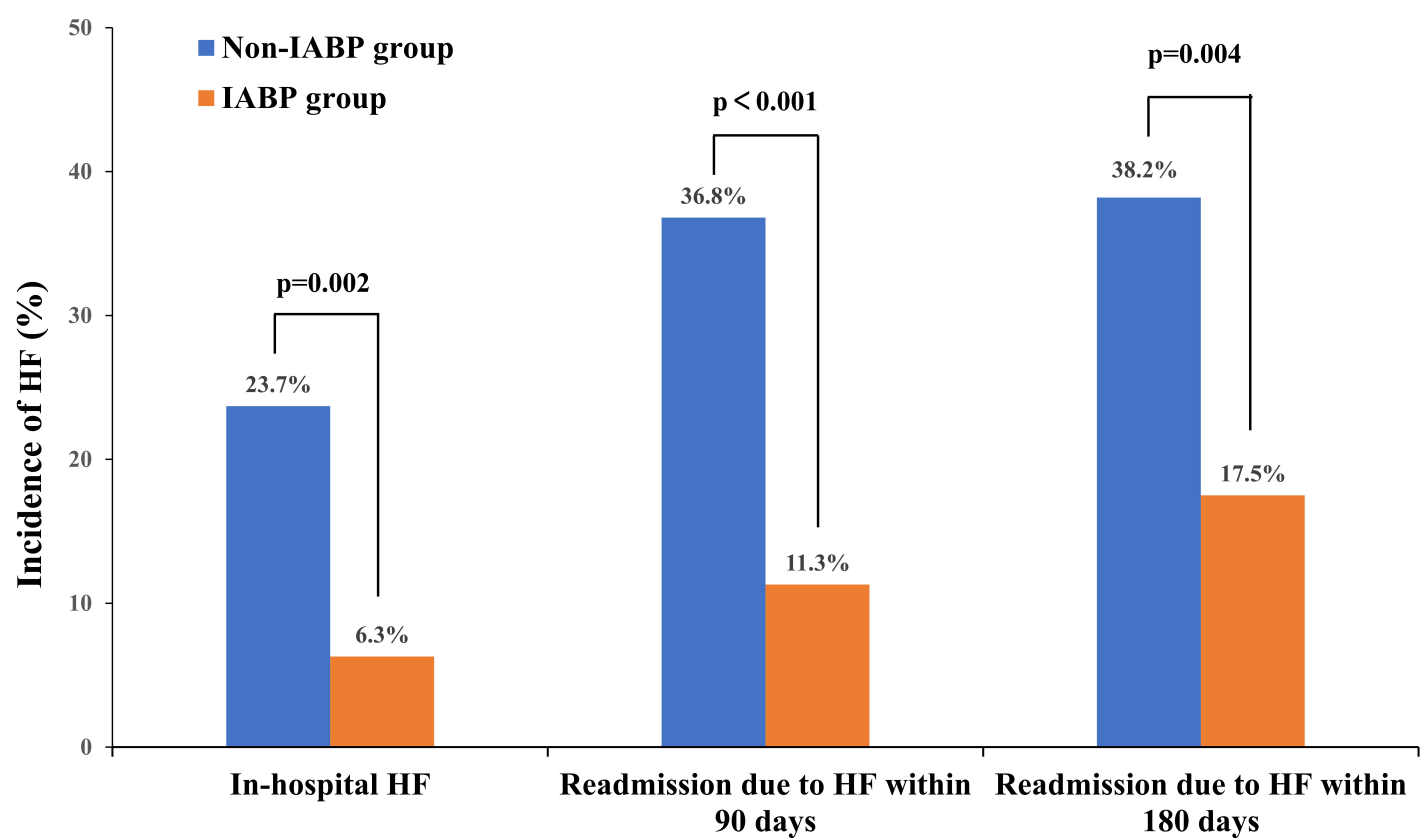
**Incidence of in-hospital HF, readmission due to HF at 90-day and 180-day intervals**. Abbreviations: IABP, intra-aortic balloon pump; HF, heart failure.

Fig. 4 summarized the incidence of in-hospital HF, readmission due to HF at 90-day and 180-day intervals.

Cox multivariate analysis was performed to investigate influential factors of readmission due to HF during the 90-day and 180-day follow-up. The analysis determined that IABP support (HR = 0.34, 95% CI: 0.15–0.76, *p* = 0.008), in-hospital HF (HR = 3.28, 95% CI: 1.29–8.36, *p* = 0.013), and periprocedural myonecrosis (HR = 4.26, 95% CI: 1.60–11.35, *p* = 0.004) were independently associated with readmission due to HF within 90-day follow up (Table 4).

**Table 4.** **Cox regression analyses of predictors for readmission due to HF within 90 days**.

Variables	Univariate cox regression analyses		Multivariate cox regression analyses	
	HR (95% CI)	*p*-value	HR (95% CI)	*p*-value
IABP implantation	0.25 (0.12–0.54)	<0.001	0.34 (0.15–0.76)	0.008
Primary RA	0.52 (0.27–0.99)	0.048	0.72 (0.37–1.39)	0.325
In-hospital heart failure	13.2 (6.75–25.76)	<0.001	3.28 (1.29–8.36)	0.013
Periprocedural myonecrosis	8.42 (4.06–17.45)	<0.001	4.26 (1.60–11.35)	0.004

HR, Hazard ratio; CI, confidence interval; HF, heart failure; IABP, intra-aortic balloon pump; RA, rotational atherectomy.

Additionally, IABP implantation (HR = 0.47, 95% CI: 0.24–0.92, *p* = 0.028), in-hospital HF (HR = 3.50, 95% CI: 1.43–8.58, *p* = 0.006), and periprocedural myonecrosis (HR = 3.20, 95% CI: 1.34–7.67, *p* = 0.009) were independent predictors of readmission due to HF during 180-day follow up (Table 5).

**Table 5.** ** Cox regression analyses of predictors for readmission due to HF within 180 days**.

Variables	Univariate cox regression analyses		Multivariate cox regression analyses	
	HR (95% CI)	*p*-value	HR (95% CI)	*p*-value
IABP implantation	0.37 (0.20–0.71)	0.003	0.47 (0.24–0.92)	0.028
Primary RA	0.47 (0.26–0.85)	0.012	0.61 (0.33–1.13)	0.113
In-hospital heart failure	11.25 (6.00–21.09)	<0.001	3.50 (1.43–8.58)	0.006
Periprocedural myonecrosis	6.14 (3.26–11.54)	<0.001	3.20 (1.34–7.67)	0.009

HR, Hazard ratio; CI, confidence interval; HF, heart failure; IABP, intra-aortic balloon pump; RA, rotational atherectomy.

## 4. Discussion

In recent years, interventional cardiologists are paying more attention to the revascularization of complex and high-risk coronary diseases. A universally agreed definition of high-risk PCI is still on debate, they may present with severely calcified, multivessel coronary disease and reduced ejection fraction (LVEF <40%). These patients are usually disqualified from CABG due to prohibitive co-morbidities including advanced age and poor cardiac function. In this subgroup, traditional PCI is a great challenge because of tough fibrocalcific and otherwise non-dilatable or non-crossable lesions, which are considered the main indication of RA. However, these patients may have significantly attenuated cardiac function reserve to withstand the procedure, because RA procedure can arise prolonged segmental left ventricle (LV) dysfunction resulting from cardiac ischemia, and then hemodynamic instability [9, 10].

Theoretically, IABP serves to rise myocardial perfusion by augmenting the coronary pressure gradient from the aorta to the epicardial coronary circulation and reducing the afterload of LV by active deflation immediately before the onset of LV systole [11, 12]. However, the role of IABP support in improving clinical outcomes of RA for complex and high-risk coronary interventions is still controversial [13, 14]. The present study investigated the impact of IABP support on in-hospital, 90-day, and 180-day outcomes after RA in patients with multivessel disease and reduced LVEF.

In the present study, all subjects were presented with high-risk and complex lesions, the IABP group had more patients with a history of pre-MI and chronic heart failure, the NT-pro BNP level was also higher, reflecting a worse cardiac function, which was the reason why more prophylactic IABP was used in these patients.

Although RA could be successfully performed in patients with impaired LV function without hemodynamic support according to Hoyle L *et al*. [14], more bailout hemodynamic support, according to the subgroup analysis, was required in the patients with impaired LV systolic function. Moreover, microvascular embolization by a large amount of debris can cause microvascular dysfunction and adversely affect the cardiac function during the RA procedure. Nevertheless, the compensation mechanism cannot be established in time and subsequently, hemodynamic compromise may occur. Therefore, patients in the present study were at high risk of hemodynamic instability since they were all presented with impaired LV systolic function (LVEF <40%). Of note, although the baseline SBP was similar, and the action mechanism of the IABP was to reduce the SBP, we observed a significantly higher SBP in the IABP group after IABP implantation. We thought that less decreasing of SBP from baseline could be the main reason for this phenomenon. As evidenced by a lower incidence of slow flow/no re-flow in the IABP group, which exactly reflecting the important role of IABP in decreasing complications and maintaining hemodynamic stability, this was consistent with the previous study [11].

Patients receiving prophylactic IABP implantation showed better in-hospital outcomes in this study. The rates of in-hospital MACE were significantly lower in the IABP group (7.5% vs. 26.3%, *p* = 0.002), and most of the MACEs were both driven by in-hospital heart failure in the two groups. There are two possible explanations for why IABP support positively affects the in-hospital prognosis in these high-risk patients. Firstly, IABP counterpulsation plays a vital role in maintaining cardiac output by reduction of the afterload (with reduced oxygen consumption and myocardial ischemia), as confirmed by a lower incidence of post-procedure hypotension in the IABP group, which may augment coronary perfusion afterwards and contribute to a decrease in ischemia [15]. Secondly, previous studies revealed that slow-flow/no-reflow during RA is mainly associated with the distal embolization of microparticulate debris [16, 17]. Since coronary blood flow occurs predominantly in diastole, IABP gives rise to the coronary pressure and increases coronary blood flow, which may hence microparticulate debris clarity and subsequently decrease the incidence of slow flow/no reflow. The present study showed a slightly lower incidence of slow flow/no reflow in the IABP group, which may decrease the risk of worsen LV function and subsequent in-hospital heart failure.

For patients who receive PCI, a low LVEF is reported to be an independent predictor of adverse cardiac events [18]. Although RA can be safely and effectively performed in patients with low LVEF with similar procedural success rates and in-hospital mortality [14], the long-term rate of MACEs was significantly higher, and low LVEF was still an independent predictor of long-term MACEs, mainly driven by HF requiring rehospitalization [19]. In our study, all patients were presented with poor LV function (LVEF <40%), and they were at high risk of morbidity and mortality. Interestingly, although patients in the IABP group had more unfavorable baseline clinical characteristics (more frequent history of MI and HF, higher-level NT-proBNP), Kaplan-Meier curves showed a significantly lower cumulative incidence of readmission due to HF in the IABP group during 90-day follow up (Log-rank test: *p* = 0.002). Besides, these benefits seemed to persist over a 180-day follow-up period. The multivariate analysis indicated that prophylactic implantation of the IABP was an independent protective factor of readmission due to HF during the 90-day and 180-day follow-up. This lasting benefit after removal of the IABP furtherly demonstrated that prophylactic use of IABP contributes to superior late clinical outcomes.

The presence of heart failure with decreased LVEF was reported as an independent predictor of mortality following RA and PCI [20, 21]. In the present study, IABP implantation before RA procedure showed a benefit of an absolute 7.1% difference in mortality during 90-day follow- up, but this difference was not statistically significant. Divaka *et al*. [6] compared the all-cause mortality after RA with IABP versus without IABP support at 6 months and found no significant difference (4.6% vs. 7.4%, *p* = 0.320), which was consistent with our findings.

## 5. Limitations

This study was a retrospective and observational analysis of data from single center with a limited sample size. There is no doubt that regularly taking medicine is of great importance for patients with CAD and HF. However, the findings from post-operational visit including regular and rational use of medicines were not available for this study, hence, it is difficult to determine the exclusive contributions of IABP to the endpoints. Nevertheless, IABP may play a vital role in maintaining hemodynamic stability during PCI with RA, especially in patients with severely calcified lesions accompanied by multivessel disease and reduced LVEF. We found that IABP was associated with reduced RA-related complications such as slow flow/no re-flow and periprocedural myonecrosis, which may partially improve short-term outcomes. In the future, prospective randomized controlled trails in a large group were needed to furtherly confirm the findings.

## 6. Conclusions

The present study suggests the important role of IABP support in improving the outcomes of patients after RA if multivessel disease and low LVEF are anticipated. Prophylactic IABP implantation was related to a lower incidence of in-hospital MACE, and readmission due to HF within 90-day and 180-day follow-up without significant impact on the procedural success and all-cause mortality.

## Data Availability

The datasets used and/or analyzed during the current study are available from the corresponding author on reasonable request.
